# Phylogenetic and Demographic Characterization of Directed HIV-1 Transmission Using Deep Sequences from High-Risk and General Population Cohorts/Groups in Uganda

**DOI:** 10.3390/v12030331

**Published:** 2020-03-18

**Authors:** Nicholas Bbosa, Deogratius Ssemwanga, Alfred Ssekagiri, Xiaoyue Xi, Yunia Mayanja, Ubaldo Bahemuka, Janet Seeley, Deenan Pillay, Lucie Abeler-Dörner, Tanya Golubchik, Christophe Fraser, Pontiano Kaleebu, Oliver Ratmann, on behalf of the MRC/UVRI & LSHTM Uganda Research Unit, The PANGEA Consortium

**Affiliations:** 1 Medical Research Council (MRC)/Uganda Virus Research Institute (UVRI) and London School of Hygiene and Tropical Medicine (LSHTM) Uganda Research Unit, Entebbe 256, Uganda; Deogratius.Ssemwanga@mrcuganda.org (D.S.); Yunia.Mayanja@mrcuganda.org (Y.M.); Ubaldo.Bahemuka@mrcuganda.org (U.B.); Janet.Seeley@mrcuganda.org (J.S.); Pontiano.Kaleebu@mrcuganda.org (P.K.); 2 Uganda Virus Research Institute, Entebbe 256, Uganda; assekagiri@uvri.go.ug; 3 Department of Mathematics, Imperial College London, London SW72AZ, UK; xiaoyue.xi16@imperial.ac.uk; 4 London School of Hygiene and Tropical Medicine (LSHTM), London WC1E 7HT, UK; 5 Africa Health Research Institute, Private Bag X7, Durban 4013, South Africa; d.pillay@ucl.ac.uk; 6 Division of Infection and Immunity, University College London, London WC1E 6BT, UK; 7 Oxford Big Data Institute, Li Ka Shing Centre for Health Information and Discovery, Nuffield Department of Medicine, Old Road Campus, University of Oxford, Oxford OX3 7BN, UK; lucie.abeler-dorner@bdi.ox.ac.uk (L.A.-D.); golubchi@well.ox.ac.uk (T.G.); christophe.fraser@bdi.ox.ac.uk (C.F.)

**Keywords:** human immunodeficiency virus, phylogenetic analysis, deep sequences, transmission, key populations

## Abstract

Across sub-Saharan Africa, key populations with elevated HIV-1 incidence and/or prevalence have been identified, but their contribution to disease spread remains unclear. We performed viral deep-sequence phylogenetic analyses to quantify transmission dynamics between the general population (GP), fisherfolk communities (FF), and women at high risk of infection and their clients (WHR) in central and southwestern Uganda. Between August 2014 and August 2017, 6185 HIV-1 positive individuals were enrolled in 3 GP and 10 FF communities, 3 WHR enrollment sites. A total of 2531 antiretroviral therapy (ART) naïve participants with plasma viral load >1000 copies/mL were deep-sequenced. One hundred and twenty-three transmission networks were reconstructed, including 105 phylogenetically highly supported source–recipient pairs. Only one pair involved a WHR and male participant, suggesting that improved population sampling is needed to assess empirically the role of WHR to the transmission dynamics. More transmissions were observed from the GP communities to FF communities than vice versa, with an estimated flow ratio of 1.56 (95% CrI 0.68–3.72), indicating that fishing communities on Lake Victoria are not a net source of transmission flow to neighboring communities further inland. Men contributed disproportionally to HIV-1 transmission flow regardless of age, suggesting that prevention efforts need to better aid men to engage with and stay in care.

## 1. Introduction

In 2019, the Joint United Nations program on HIV/AIDS (UNAIDS) reported an estimated 1.4 million people that were living with HIV-1 in Uganda [1]. Despite significant decreases in HIV-1 prevalence and incidence since the early 1990s, the epidemic in Uganda remains self-sustaining through heterosexual transmission in the general population (GP) [2]. Certain key populations in Uganda are particularly affected by HIV-1. These include fisherfolk (FF), women at high risk of HIV-1 infection (WHR), men who have sex with men, injection drug users, as well as men and women of the uniformed forces [3]. Average national HIV-1 incidence is estimated at less than 1 per 100 person years at risk (1/100 PYAR) [4], while incidence rates among FF and WHR are respectively estimated at 6/100 PYAR [5] and 3/100 PYAR [6]. Individuals in these groups also have a higher tendency to engage in high-risk sexual behavior, which led to the hypothesis that they may drive HIV-1 transmission in general, and sustain transmission in the GP [2,7].

UNAIDS and the WHO have recommended that countries develop combination HIV-1 intervention packages that are tailored to their high-risk/key populations in order to curb the HIV-1 epidemic in a cost-effective manner [8,9]. To achieve this, several strategies have been suggested. These include spatial mapping to identify geographic areas of higher-than-average HIV-1 prevalence [10] and demographic surveillance to characterize populations that are at higher risk of HIV-1 infection [11,12]. Recent studies in Uganda have employed spatial mapping and demographic surveillance prior to the implementation of combination interventions in high-risk populations like FF [13,14] and WHR [15]. In addition, phylogenetic analyses have been increasingly used to characterize aspects of HIV-1 transmission dynamics from nucleotide sequences of the virus. For instance, phylogenetic analysis helped to reconstruct the historical spread of HIV-1 [16,17,18], characterize HIV-1 transmission networks [19,20], identify traits associated with onward viral transmission in high-risk groups [21,22], and evaluate HIV-1 migration patterns [23,24]. In combination with sociodemographic or epidemiological data, these approaches have provided useful insights in assessing risk factors associated with HIV-1 spread [25] and identifying micro-epidemics [26], thus informing prevention strategies. Furthermore, they have been applied in source-attribution studies to assess the role of viral introductions from other communities [27,28], and to identify populations that serve as sinks or sources for HIV-1 transmission [29,30]. In particular, we previously estimated that high-prevalence FF communities located in central and southwestern Uganda are net recipients (sinks) of local HIV-1 transmission flow, based on phylogeographic analysis of 208 subtype A and 177 subtype D consensus sequences [29]. A second analysis estimated that there were 2.50 times (95% credibility interval (95%CrI) 1.02–7.30) more transmissions from inland to fishing areas than vice versa, based on a sample of 2652 viral deep-sequences and after accounting for human migration dynamics [30]. The two studies thus showed that the relatively small FF communities around Lake Victoria are not driving the surrounding HIV-1 epidemics regardless of their high HIV-1 prevalence.

**Figure 1:**
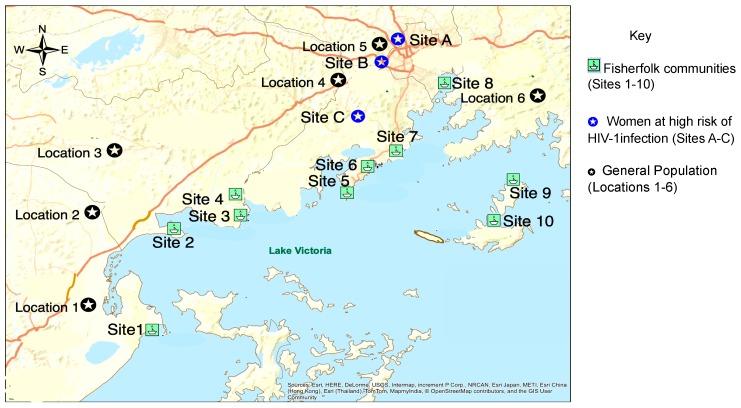
Map showing the sampling sites. Sites 1 to 10 show the fisherfolk (FF) communities and sites A to C show areas where women at high risk of HIV-1 infection (WHR) and some of their clients were enrolled. Locations 1 to 6 show areas in the general population (GP) where study participants were enrolled. Almost half (40.9%) of HIV-1 positive participants enrolled in clinics and study sites (see Table 1) shown on the map in south central Uganda had their samples deep sequenced and subjected to phylogenetic analyses.

Here, we test the finding that FF communities are net sinks and not sources of local transmission flow on an independent data set that includes a larger set of fishing villages. In addition, we aim to assess the extent to which key populations (FF and WHR) and socio-demographic factors contribute to the HIV-1 epidemic in the GP. To this end, the Medical Research Council/Uganda Virus Research Institute and London School of Hygiene & Tropical Medicine (MRC/UVRI and LSHTM) Uganda Research Unit team enrolled HIV-1 infected individuals in fishing villages along the shores of Lake Victoria, neighboring inland areas (approximately 10–40 km) and hotspots of women engaged in high-risk sexual behavior (see Figure 1 under Materials and Methods). The PANGEA (Phylogenetics And Networks for Generalized Epidemics in Africa) consortium (Appendix A and Appendix B) [31] generated HIV-1 deep-sequences from this convenience sample of infected individuals. The deep-sequence data was then used to reconstruct viral phylogenetic transmission networks that contained information on the direction of transmission. Pairs with phylogenetically highly supported evidence for the direction of transmission were identified in the networks, and these pairs were used to quantify HIV-1 transmission flows between the GP, FF, and WHR populations. In doing so, this study also demonstrates that HIV deep-sequence data can now be routinely used to reconstruct HIV-1 transmission networks, and provide important information on the direction of transmission within them.

## 2. Materials and Methods

### 2.1. Study Design and Population

The MRC/UVRI and LSHTM Uganda Research Unit (MRC) conducted several population-based cross-sectional surveys in HIV-1 high-risk and general population groups in central and southwestern Uganda [29,32,33,34,35,36]. In this analysis, we combined data collected from several populations surveyed between August 2014 and August 2017. Figure 1 below shows a map of the study sites.

The FF study population included individuals from 10 FF communities [13,19,37,38,39] as shown in Figure 1. Individuals were approached in community health facilities (CHF) or during voluntary counselling and testing (VCT) campaigns, and were eligible to participate if they had an HIV-1 positive status and were at least 18 years old (FF communities 1–8) or 14 years (FF communities 9–10). 

The WHR study population included participants of the MRC Good Health for Women’s Project (GHWP), and an extension of this study population to other commercial sex work areas (non-GHWP). The GHWP cohort was initiated in 2008, when initially 1027 women were recruited with the objective of studying the size, determinants, and dynamics of the HIV-1 epidemic, and remains an open cohort with approximately 3000 women enrolled to-date [6,40]. Women above 18 years of age who reported being involved in commercial sex work and/or high-risk sexual behavior, and their clients who consented to participate were enrolled in the study. Data were collected on socio-demographic characteristics and risk behavior, and consenting participants were tested for HIV-1.

The GP study population included individuals from rural communities surveyed through the MRC General Population Cohort (GPC) since 1989 [33,41]. We additionally sampled areas that neighbor the FF populations, as well as areas that are geographically close to the WHR recruitment area. In these communities, individuals were recruited in CHF or during VCT campaigns, and eligible if they were HIV-1 positive and were at least 16 years old. Further, individuals with HIV-1 infection who received care or were diagnosed through VCT were invited to participate. Participants completed structured questionnaires that captured general demographic, socioeconomic, marital, partnership histories and behavioral data. A biometric fingerprint-scanning device was used on all study participants to avoid duplicate enrolments [42].

### 2.2. Ethics Statement

This study was part of the MRC HIV-1 Molecular Epidemiology study which was approved by the Uganda Virus Research Institute Research and Ethics Committee (UVRI-REC) on the 24th/06/2013 (Federal Wide Assurance (FWA) No. 00001354, Project identification code: GC/127/13/06/27) and the Uganda National Council for Science and Technology (UNCST) on the 12th/09/2013 (FWA No. 00001293, Project identification code: HS 1432). All participants were recruited voluntarily and provided written informed consent. 

### 2.3. Deep Sequencing and Assembly of HIV-1 Reads

First, 10 mL of blood was collected from the study participants by venipuncture, including those on antiretroviral therapy (ART). Next generation sequencing was performed on samples from infected participants who did not report ART use and who had sufficient plasma viraemia (viral load >1000 copies/mL). HIV-1 deep sequencing was performed using the Illumina Miseq platform at the Africa Health Research Institute (Durban, South Africa), and at the Wellcome Trust Sanger Institute (Hinxton, UK) [43,44]. Briefly, viral RNA was extracted from plasma samples using the QIAamp Viral RNA Minikit (Qiagen Inc., Valencia, CA, USA). Reverse transcription and amplification of the extracted viral RNA was done in a one-step RT-PCR reaction using a set of 4 HIV-1 primers that were designed for the amplification of HIV-1 genomes in four overlapping amplicons for all groups and subtypes [43]. Amplicons were pooled in equimolar quantities according to the manufacturer’s instructions for the library preparation step using the Nextera XT kit. Library validation was performed using the Agilent Bioanalyzer before sequencing was done using the Miseq 250-bp paired end technology as previously described [43]. Deep-sequencing reads were assembled using the Shiver pipeline [45]. 

### 2.4. Deep-Sequence Phylogenetic Analysis

**Figure 2:**
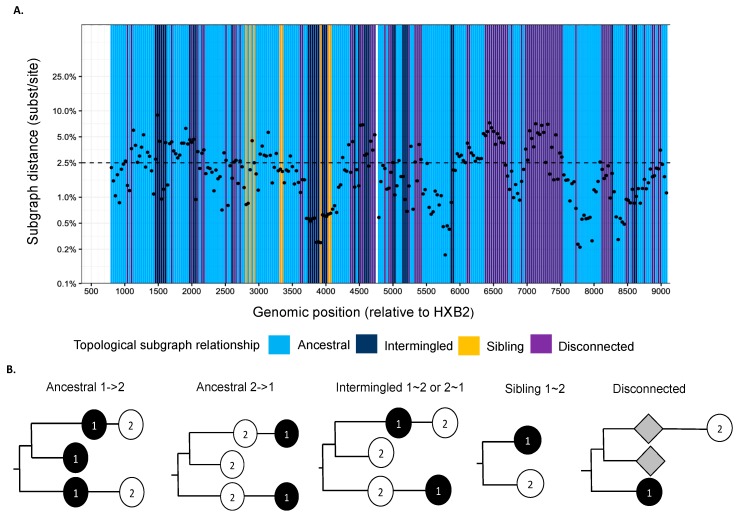
(**A**) Phyloscan plot showing deep-sequence phylogenies reconstructed at 250 bp genomic window intervals across the genome. Ancestral state reconstruction was used to attribute phylogenetic lineages to individuals, and phylogenetic subgraphs were defined as groups of lineages that were attributed to the same individual. For a pair of individuals, the scan plots show the shortest patristic distance between subgraphs of both individuals (*y*-axis) and the topological relationship between subgraphs of both individuals (colors) across the genome (ancestral to one another (light blue), siblings (yellow), intermingled (dark blue), or disconnected (purple)). (**B**) Description of topological relationship between subgraphs in deep-sequence phylogenies. The phylogenetic topology or ordering of subgraphs is used to infer the likely direction of transmission between individuals [48]. Ancestral 1->2 means that at least one subgraph from individual 1 is phylogenetically adjacent to a subgraph from individual 2, and that all adjacent subgraphs from individual 1 are ancestral to those of individual 2. This phylogenetic pattern is consistent with transmission from individual 1 to individual 2, possibly via unsampled intermediates. Similarly, ancestral 2->1 means at least one subgraph from individual 1 is phylogenetically adjacent to a subgraph from individual 2, and that all adjacent subgraphs from individual 1 are descendent from those of individual 2. This phylogenetic pattern is consistent with transmission from individual 2 to individual 1, possibly via unsampled intermediates. Intermingled 1~2 means that at least one subgraph from individual 1 is phylogenetically adjacent to one subgraph from individual 2, and that there are adjacent subgraphs from individual 1 ancestral to those of individual 2, and vice versa. Sibling 1~2 means that at least one subgraph from individual 1 is phylogenetically adjacent to one subgraph from individual 2, and that the adjacent subgraphs are phylogenetically next to each other. These two patterns indicate that the two individuals are closely related, but there is no evidence for the direction of transmission. Disconnected refers to individuals who have subgraphs that are not adjacent to each other. This pattern suggests that the two individuals are phylogenetically unlinked.

We used phyloscanner version 1.8.0 to reconstruct directed HIV-1 transmission networks from deep sequences [46]. Briefly, phyloscanner analysis starts with HIV deep-sequence reads from a set of infected individuals that are mapped against reference sequences of the entire genome. Across the genome, sliding windows are defined, and for each window, paired-end reads from all individuals that overlap the window are extracted and aligned, and deep-sequence phylogenies reconstructed. In our analysis, windows were of length 250 bp and incremented by 25 bp across the entire genome. Next, viral lineages in the deep-sequence phylogenies were attributed to each individual using maximum parsimony ancestral state reconstruction, which defined larger parts (called “subgraphs”) of the deep-sequence phylogenies that are attributed to viral evolution within each individual. Using Sanger sequencing, typically one sequence is available per person, and in this case, each subgraph consists of the tip sequence only that corresponds to the sampled individual. Using deep-sequence data, subgraphs of each individual typically comprise several tips and inner nodes in the phylogeny, and this information can be used to characterize the relationship of individuals in terms of distance between subgraphs and topological relationship between subgraphs. Figure 2 shows the subgraph distances (*y*-axis) and subgraph topologies (color) between a pair of individuals in the phylogenies that were reconstructed from the overlapping 250 bp read alignments across the genome. The subgraph distances are used to infer phylogenetic linkage, and the subgraph topologies are used to infer the likely direction of transmission between individuals (Figure 2b). A phyloscanner analysis returns for each pair of individuals phylogenetic scores for linkage and transmission direction that are calculated across all genomic windows [46,47,48]. The scores indicate the proportion of genomic windows with phylogenetic support for linkage and transmission direction, after accounting for overlap between the read alignments.

### 2.5. Reconstruction of Transmission Networks

To reconstruct phylogenetic transmission networks, we first determined the deep-sequence phylogenetic relationships between all possible pairs of individuals in the sample with phyloscanner. Analysis proceeded in batches of 75 individuals, which considerably reduced the number of deep-sequence phylogenies that had to be estimated, and computational burden [47]. Second, from this output we identified pairs of individuals between whom phylogenetic linkage could not be excluded based on subgraph distance in deep-sequence phylogenies. Third, potentially phylogenetically linked pairs of individuals were grouped into larger phylogenetic transmission networks so that each individual was linked to at least one other individual. Fourth, each potential transmission network was separately analyzed with phyloscanner to substantiate membership of individuals in networks, and to estimate the phylogenetically likely direction of viral spread within networks. Fifth, we considered all possible transmission chains in these networks, which corresponded to all possible minimum (directed) spanning trees, similar as in [49,50]. We then identified the most likely transmission chain as the minimum spanning tree that maximized the product of phyloscanner transmission scores in the directions of the edges in the spanning tree [47]. Sixth, we extracted pairs of individuals with strong support for phylogenetic linkage and strong support for the direction of transmission in the phylogenetically most likely transmission chain (source–recipient pairs), using a threshold of 0.6 on the phyloscanner scores (see [47] for a justification of the threshold). The analysis protocol and phyloscanner runtime arguments were exactly as described in [47]. This analysis in general cannot rule out the presence of unsampled intermediates in the phylogenetically most likely transmission chains, and among source–recipient pairs.

### 2.6. Quantifying HIV-1 Transmission Flows

We used the phyloflows package, version 1.1.0, implemented in the R software [51] to quantify HIV-1 transmission flows within and between the three populations, while adjusting for sampling heterogeneity across our study populations. Briefly, a Bayesian model was used to estimate transmission flows in population groups under the assumption that each group was sampled at random, but with potentially different sampling rates [30]. A Markov Chain Monte Carlo (MCMC) algorithm was used to obtain samples from the posterior distribution of transmission flows, was run for 50,000 iterations, and the initial 5% were discarded as burn-in. Convergence was assessed with the Gelman–Rubin statistic [52], and all effective sample sizes of the marginal posterior transmission flows were well above 1000. Examplary MCMC outputs are shown in Supplementary Figure S1.

## 3. Results

### 3.1. Population-Based Sample of HIV-1 Deep Sequences

Between August 2014 and August 2017, a total of 6185 HIV-1 positive individuals were enrolled in CHF and VCT campaigns, of whom 2531 were deep-sequenced (at a sequencing depth of >30× over at least 5000 nt of the HIV-1 genome). A total of 3200 participants were from the GP of whom 1578 were male and 1622 female. Then, 1309 (40.9%) samples were deep-sequenced, comprising 636 men (40.3% of 1578) and 673 women (41.5% of 1622). Further, 2185 participants were from fishing sites of whom 1103 were male and 1082 were female. Then, 895 (41.0%) samples were deep-sequenced, comprising 468 men (42.4% of 1103) and 427 (39.5% of 1082) women. There were 720 women at high risk and 80 male clients recruited, of whom 301 (41.8%) women at high risk and 26 (32.5%) clients were deep-sequenced. Table 1 provides further demographic information on the recruited and sampled individuals. The proportion of men and women who had their samples deep sequenced in each study population were similar, with the exception of male clients of women at high risk.

### 3.2. Reconstructed HIV-1 Transmission Networks

**Figure 3:**
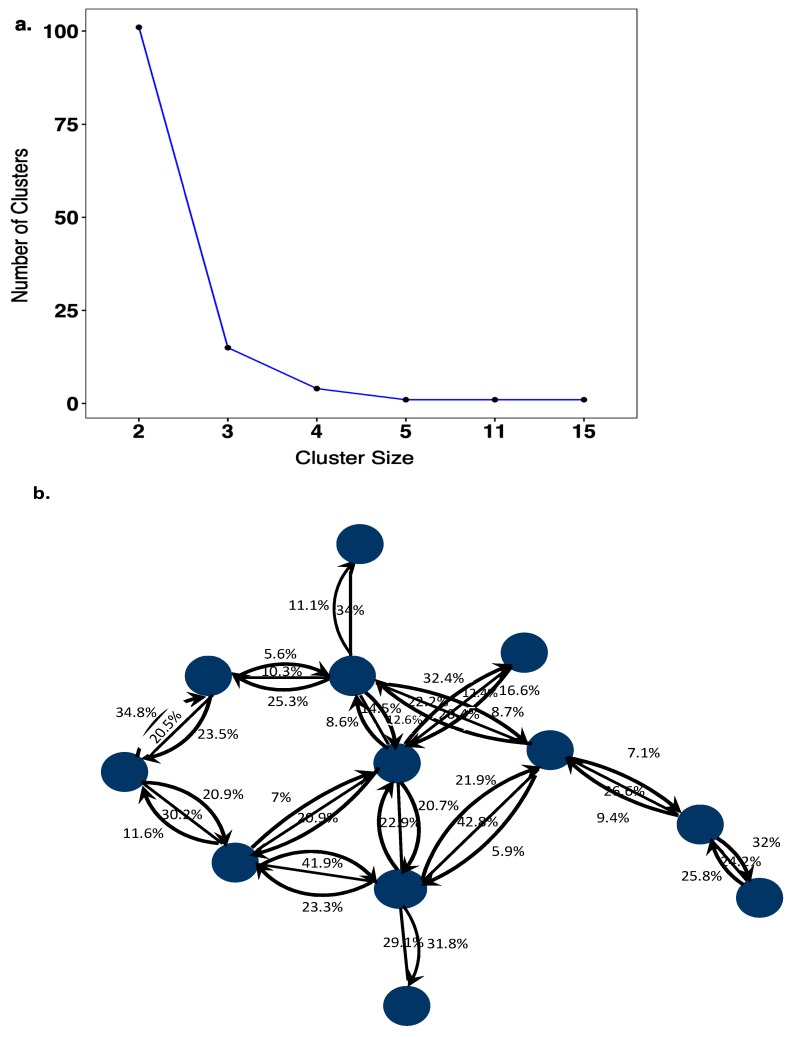
(**a**) Cluster size distribution of HIV-1 transmission networks. The cluster size distribution of transmission networks consisted of 101 sequences that were linked as pairs, 15 clusters of 3 linked sequences, 4 clusters of 4 linked sequences, 1 cluster of 5 linked sequences, 1 cluster of 11 linked sequences and 1 cluster of 15 linked sequences. (**b**) Transmission pairs with phylogenetic evidence for the direction of HIV-1 transmission. The blue colored circles represent sequences that are linked to one another with phyloscanner scores (%) showing support for transmission in either direction between individual sequences. Source–recipient pairs that had phyloscanner scores >60% for linkage and >60% for one direction were considered highly supported.

A total of 123 HIV-1 transmission networks were reconstructed with phyloscanner, of which 101 (82.1%) comprised of 2 individuals, 20 (16.3%) comprised of 3–5 individuals, 0 comprised 6–10 individuals, and 2 (1.6%) comprised of >10 individuals (Figure 3a). In larger networks, pairs of individuals between whom linkage was not excluded had larger subgraph distances (median distances were 0.5% in networks of size 2, 0.6% in networks of size 3–5, and 2.1% in networks of size >10; see Table S1), suggesting that in larger networks, slightly more phylogenetic distant individuals were connected. In the reconstructed transmission networks, a total of 105 source–recipient pairs with strong support for phylogenetic linkage and direction of transmission were identified based on the criteria described under methods. Further analysis was restricted to these highly supported source–recipient pairs. Figure 3b illustrates one of the largest identified networks. In the figure, source–recipient pairs are highlighted with arrows in black. No arrows were drawn when the corresponding two individuals were classified as phylogenetically unlinked in at least 60% of deep-sequence phylogenies across the genome.

### 3.3. HIV-1 Transmission within and between Study Participants of the General Population, Fisherfolk, and the Women at High Risk Cohort and their Clients

The phylogenetically reconstructed networks included 105 source–recipient pairs (Table 2, column three). The majority of reconstructed source–recipient pairs were within the population groupings. Thirty-six (34.3%) were from GP to GP; 33 (31.4%) were from FF to FF, and 10 (9.5%) were from WHR to WHR. Between population groups, we found 14 (13.3%) source–recipient pairs from GP to FF, 9 (8.6%) pairs from the FF to the GP, 2 (1.9%) pairs from FF to WHR, and 1 pair (1.0%) from WHR to the GP. 

Considering gender, 50 (47.6%) source–recipient pairs were male to female, 12 (11.4%) were male to male, 24 (22.9%) were female to male, 10 (9.5%) were female to female among WHR, and 9 (8.6%) were female to female and involved at most one WHR (Supplementary Table S2). Observing male–male pairs was not significantly more likely than observing female–female pairs outside WHR. Thus, the most likely explanation for the presence of phylogenetically linked same-sex pairs is that intermediate individuals or common source cases of the opposite gender remained unsampled. In particular, there was only 1 male–female pair among the 13 highly supported source–recipient pairs that involved WHR, suggesting that a substantial number of WHR male clients were not captured in our populations. Due to this under-sampling of male clients, pairs from the WHR population were excluded from the subsequent HIV-1 transmission flow analysis. Column four in Table 2 lists the phylogenetically reconstructed source–recipient pairs after excluding same-sex pairs. 

We estimated HIV-1 transmission flows within and between the FF and GP study participants, while accounting for slightly different sequence sampling fractions of HIV-1 positive participants. Overall, transmission was assortative within the FF and GP participants, with an estimated assortativity coefficient of 0.32 (95% CrI 0.10–0.53). More specifically, the estimated transmission flows when same-sex pairs were included are listed in Table 2 column five, and the estimated transmission flows when same-sex pairs were excluded are listed in Table 2 column six. Within and between groups of study participants, the estimated HIV-1 transmission flows were 45.5% (95% credibility interval (CrI) 34.1–57.0%) from FF to FF, 12.7% (95% CrI 6.1–21.3%) from FF to GP, 19.5% (95% CrI 11.4–29.3%) from GP to FF, and 22.3% (95% CrI 13.4–32.7%) from GP to GP in the analysis excluding same-sex pairs. The estimated flows were significantly different when same-sex pairs were excluded, because 20 (95.2%) of 21 same-sex source–recipient pairs were within the GP. All phylogenetically reconstructed transmissions between GP and FF locations involved a male and a female, and so there were more transmissions from GP to FF locations compared to the opposite direction regardless of excluding same-sex source–recipient pairs. The estimated flow ratio of transmission flows from GP to FF divided by flows from FF to GP was 1.56 (95% CrI 0.68–3.72).

We next estimated the sources of HIV-1 acquisition among the FF and GP study participants after adjusting for sequence sampling differences (Table 3). For any HIV-1 infection found in the FF sample, the probability that it originated from other FF was 70.4% (95% CrI 56.2–81.9%), and the probability that it originated from the GP was 29.6% (95% CrI 18.1–43.8%) when same-sex pairs were excluded. For any HIV-1 infection found in the GP sample, the probability that it originated from the GP was 80.2% (95% CrI 66.8–90.0%), and the probability that it originated from the FF was 19.8% (95% CrI 10.0–33.2%) when same-sex pairs were excluded. 

### 3.4. HIV Transmission by Gender

After excluding same-sex pairs and accounting for differences in sequence sampling fractions of men and women, an estimated 68.0% (56.6–78.1%) of transmissions in the sample originated from men and 32.0% (21.9–43.4%) from women.

### 3.5. HIV-1 Transmission by Age Groups

We further characterized the age-specific HIV-1 transmission flows among the phylogenetically highly supported source–recipient pairs (Table 4). We sought to use the same age band as in de Oliveira et al. [25] to enable a comparison between the population sampled in KwaZulu-Natal, South Africa, and our settings in central and southwestern Uganda. However, with 74 reconstructed source–recipient pairs between men and women, we reduced these further to age bands 18–24 years and 25–59 years at time of diagnosis. In the sample, transmission was not assortative within the two age groups in both the male-to-female and female-to-male direction, with estimated assortativity coefficients of 0.07 (95% CrI −0.18–0.33) and −0.08 (−0.40–0.28) respectively. More detailed analysis of the transmission flows showed that most (*n* = 18, 25.0%) phylogenetically reconstructed transmissions occurred from men of 25–59 years to women of 25–59 years, followed by transmissions (*n* = 13, 18.1%) from men of 25–59 years to women of 18–24 years. The estimated transmission flows among participants after adjusting for sequence sampling differences are reported in Table 4 column 4. Considering transmission flows from men to women compared to vice versa, the estimated flow ratios were 3.16 (95% CrI 0.92–14.44) from men aged 18–24 years to women aged 18–24 years compared to vice versa, 1.29 (95% CrI 0.47–3.69) from men aged 18–24 years compared to vice versa, 2.63 (95% CrI 0.96–8.28) from men aged 25–59 compared to vice versa, and 2.29 (95% CrI 1.02–5.52) from men aged 25–59 to women aged 25–59 compared to vice versa. 

We also estimated the sources of infection among women aged 18–24 years and women aged 25–59 years in our sample, and similarly for men (Table 5). Considering women aged 18–24 years in the sample, an estimated 34.7% (17.1–55.9%) of infections originated from men aged 18–24 years, and 65.3% (95% CrI 44.1–82.9%) of infections originated from men aged 25–59 years. Considering women aged 25–59 years in the sample, an estimated 27.7% (14.1–45.7%) of infections originated from men aged 18–24 years, and 72.3% (54.3–85.9%) of infections originated from men aged 25–59 years. Considering men aged 18–24 years in the sample, an estimated 24.2% (6.3–54.0%) of infections originated from women aged 18–24 years, and 75.8% (46.0–93.7%) of infections originated from women aged 25–59 years. Considering men aged 25–59 years in the sample, an estimated 32.6% (12.7–59.9%) of infections originated from women aged 18–24 years, and 67.4% (40.1–87.3%) of infections originated from women aged 25–59 years.

## 4. Discussion

In this study, we reconstructed phylogenetic transmission networks from HIV-1 deep sequences in a large convenience sample of HIV-1 infected individuals from high-risk and general population groups in central and southwestern Uganda. Based on the reconstructed transmission networks, we estimated HIV-1 transmission flows within and between distinct populations (GP, FF, and WHR) in our sample, and by gender and age. We also estimated the sources of HIV-1 infection in these population groups. Several molecular epidemiology studies have used nucleotide sequence data generated by Sanger sequencing to characterize HIV-1 transmission dynamics in FF communities [19,53] and other high-risk groups [23]. Here, we were able to infer directed HIV-1 transmission networks through viral phylogenetic analysis, because deep-sequencing output generated multiple sequence reads from each sampled individual, from which the direction of transmission could be inferred at an accuracy sufficient for population-level analyses [46,48]. We estimated a higher proportion of HIV-1 transmissions among participants from the FF (45.5% (34.1–57.0%) compared to participants from the GP (22.3% (13.4–32.7%) after adjusting for heterogeneity in the proportion of participants that were sequenced. These estimates do not necessarily imply that a larger proportion of transmissions occur in FF populations compared to GP populations, because the fraction of the FF populations surveyed was larger than the fraction of the GP populations surveyed. However, sampling fractions cancel out in the flow ratio statistics. Specifically, if $\pi_{ij}^{0}$ denotes the true transmission flows from population group i to population group j and $\pi_{ij}^{r}$ the transmission flows among recruited individuals of groups i and j, then:

$$\frac{\pi_{ij}^{r}}{\pi_{ji}^{r}}=\frac{p_{i}^{r}p_{j}^{r}\pi_{ij}^{0}}{p_{i}^{r}p_{j}^{r}\pi_{ji}^{0}}=\frac{\pi_{ij}^{0}}{\pi_{ji}^{0}},$$

where $p_{i}^{r}$ denotes the probability of recruiting an infected individual from population group i into the convenience sample. This equation assumes that individuals were equally likely to be recruited whether they were a source of further infections or not. Thus, the flow ratios that we inferred in our convenience sample can be interpreted as estimates of the corresponding population flow ratios. We found that the flow ratio of transmissions from GP to FF compared to vice versa was 1.56 (95% CrI (0.68–3.72). Although not statistically significant, our inferences provide further evidence that high-prevalence fishing communities along Lake Victoria are a sink and not a source of the local epidemic [29,30].

We found a substantial number of viral introductions into FF communities. Considering that participants were disproportionally recruited in FF populations, we expect that the estimated proportion of GP infections in the sample attributable to GP sources (80.2%) is a lower bound of the proportion of infections in the GP population that is attributable to GP sources. Similarly, we expect that the estimated proportion of FF infections in the sample attributable to FF sources (70.4%) is an upper bound of the proportion of infections in the FF population that is attributable to FF sources. In a study done in Rakai [30], differences in survey sampling were adjusted for, and the estimated proportion of FF infections attributable to FF sources was 54.8% (95% CrI 42.2–69.0%). Thus, taken together, the two studies indicate very high levels of viral introductions to the HIV-1 epidemic in FF populations for the duration of both studies, from August 2011 to August 2017.

HIV-1 transmission in the GP and across populations into the FF communities was mostly driven by individuals above 25 years of age relative to those younger than 25 years. We found that an estimated 65.3% (44.1–82.9%) of women aged 18–24 years in the sample were infected by men above 25 years, consistent with findings from a recent study conducted in South Africa [25]. However, we also found that an estimated 75.8% (46.0–93.7%) of men aged 18–24 years in the sample were infected by women above 25 years. There was no combination of age groups in which transmissions from women to men outweighed those from men to women, suggesting that women in the sample are “sinks” of HIV-1 transmission regardless of age. These observations challenge the concept of a transmission cycle wherein young women are infected by older men, and then transmit to men of similar age [25].

This study has limitations. First, individuals were recruited in health care facilities and during VCT campaigns, and so this study did not include infected individuals who had not yet linked to care. Second, our convenience sample preferentially included infected individuals from FF populations, rendering it challenging to extrapolate transmission flow estimates from study participants to the population the participants were recruited from. However, the flow ratio statistics in Table 2 and Table 4 are invariant to differential sampling of population groups, and can thus be used to characterize population-level HIV-1 spread. Third, few WHR male clients were recruited, and we were not able to substantiate the transmission probabilities from this population as a source of infections. Recruitment of male clients and partners of WHR in the parent study was challenging due to stigma or other unknown socio-behavioral factors that the men in this group associated with study participation. We observed from our socio-demographic data that WHR were a mobile group of individuals who interacted with high-risk male individuals in the GP and FF particularly during peak seasons of high fish stock, suggesting that a non-negligible proportion of infections may originate from WHR. Fourth, virus from less than 50% of enrolled participants could be deep-sequenced, excluding those that did not have sufficient viraemia for HIV-1 nucleic acid amplification and deep sequencing. Our sample comprised study participants who were ART naïve or on therapy with unsuppressed virus, and it is possible that transmissions from individuals who reached viral suppression soon after infection are under-represented in our data. Generation of additional HIV-1 deep sequence data based on sequencing protocols that amplify proviral HIV-1 DNA or sequence virus from low viraemic specimens [54,55] is a consideration for our future analyses. Fifth, transmissions including individuals with co-infections may have been excluded by the definition of source–recipient pairs. It is important to note that while the FF communities investigated in this study were net recipients for viral transmission (sinks) from GP population groups, the possibility of fishing villages being sources of HIV-1 transmission to other populations cannot be ruled out.

Phylogenetic reconstruction of HIV transmission networks is increasingly common to characterize HIV dynamics, typically based on tools such as HIV-TRACE [56] or Cluster Picker [57]. These tools can be applied to Sanger sequence data, and the reconstructed networks represent sets of phylogenetically linked individuals whose viral genetic relatedness suggest a direct or indirect epidemiologic link. In contrast, the deep-sequence approach adopted here provides further information into the direction of transmission between individuals in such networks using additional signal in the topology of deep-sequence phylogenetic trees. However, while the deep-sequence approach is advantageous in several aspects, there are important potential limitations to consider in future applications. Most obviously, deep-sequence phylogenetic analyses require deep-sequence data, which is only beginning to become more broadly available [31,58,59]. It is also important to consider the substantially larger requirements on storage (on the order of hundreds of gigabytes for our population-based study), as well as analysis time (~300 phylogenies reconstructed for each potential transmission network across sliding windows of the entire HIV genome is computationally intensive). Finally, due to the complexity involved in handling deep-sequence data, available analysis software does not easily install on Windows systems [46,60]. However, these challenges are primarily of logistic nature and we see no fundamental reason that prevents use of HIV deep-sequence technology to support the End the HIV initiative in the US as well as similar efforts [61,62]. 

In conclusion, the reconstructed patterns of HIV-1 transmission found across cohorts of HIV-1 infected individuals in central and southwestern Uganda support previous findings [29,30] that high-prevalence fishing communities on Lake Victoria are net recipients of HIV-1 transmission flow in the local epidemic, and not a net source of transmission flow to the neighboring low-prevalence communities located further inland. We suggest that geographically targeted interventions that focus on high-prevalence FF populations should not restrict the roll-out of preventative measures in neighboring areas that could be sources of HIV-1 infection. Furthermore, this study supports calls to re-design prevention interventions to more effectively aid men of all ages to engage with and stay in care, and thereby reduce the disproportionate infection burden among women. Two-pronged intervention approaches that target both viral transmission sources to reduce transmission and sink populations to reduce risk of infection appear optimal in reducing the future HIV-1 incidence in key and general population groups.

## Figures and Tables

**Table 1:** Study population, and proportion of sampled and sequenced individuals.

Population and Gender	HIV-Positive Participants	Deep Sequenced	Proportion of HIV-Positive Participants Who Were Sequenced
**Total**	**6185**	**2531**	**40.9%**
**Fisherfolk**	**2185**	**895**	**41.0%**
Men	1103 (50.5%)	468 (52.3%)	42.5%
Women	1082 (49.5%)	427 (47.7%)	39.5%
**General population**	**3200**	**1309**	**40.9%**
Men	1578 (49.3%)	636 (48.6%)	40.3%
women	1622 (50.7%)	673 (51.4%)	41.5%
**Women at high risk and male clients**	**800**	**327**	**40.9%**
Men	80 (10.0%)	26 (8.0%)	32.5%
Women	720 (90.0%)	301 (92.0%)	41.8%

**Table 2:** Phylogenetically observed HIV-1 transmission flows.

Sources	Recipients	Phylogenetically Strongly Supported Transmission Pairs Including Same-Sex Pairs * (Count, Proportion)	Phylogenetically Strongly Supported Transmission Pairs Excluding Same-Sex Pairs * (Count, Proportion)	Estimated Transmission Flows among Study Participants, Based on Data Including Same-Sex Pairs ** (Mean, 95% Credibility Interval of Posterior Density)	Estimated Transmission Flows among Study Participants, Based on Data Excluding Same-Sex Pairs ** (Mean, 95% Credibility Interval of Posterior Density)	Estimated Transmission Flow Ratios, Based on Data Excluding Same-Sex Pairs *** (Mean, 95% Credibility Interval of Posterior Density)
FF	FF	33 (31.4%)	33 (44.6%)	35.8% (26.2–46.1%)	45.5% (34.1–57.0%)	--
FF	GP	9 (8.6%)	9 (12.1%)	9.9% (4.7–16.8%)	12.7% (6.1–21.3%)	--
FF	WHR	2 (1.9%)	1 (1.4%)	--	--	--
GP	FF	14 (13.3%)	14 (18.9%)	15.3% (8.7–23.3%)	19.5% (11.4–29.3%)	1.56 (0.68–3.72)
GP	GP	36 (34.3%)	16 (21.6%)	39.0% (29.0–49.4%)	22.3% (13.4–32.7%)	--
WHR	GP	1 (1.0%)	1 (1.4%)	--	--	--
WHR	WHR	10 (9.5%)	0 (0%)	--	--	--

* Phylogenetically reconstructed transmission events, unadjusted for sequence sampling differences across populations. ** Estimates were obtained with the phyloflows source attribution model, are based on the number of phylogenetically reconstructed events, and are adjusted for sequence sampling differences across populations. Flows from and to WHR were not estimated due to under-sampling of WHR clients, see main text. *** Estimated flow ratios were GP->FF / FF->GP.

**Table 3:** Phylogenetically estimated sources of HIV-1 acquisition among FF and GP study participants.

	Estimated sources of infection among study participants, based on data excluding same-sex pairs * (mean, 95% credibility interval of posterior density)	
	FF	GP
**Recipient**		
FF	70.4% (56.2–81.9%)	29.6 (18.1–43.8%)
GP	19.8% (10.0–33.2%)	80.2% (66.8–90.0%)

* Estimated transmission probabilities from a source to recipient location; shown are the mean and corresponding 95% credibility interval of posterior density.

**Table 4:** Phylogenetically estimated transmission flows by age.

		Phylogenetically strongly supported transmission pairs excluding same-sex pairs * (count, proportion)	Estimated transmission flows among study participants, based on data excluding same-sex pairs ** (mean, 95% credibility interval of posterior density)	Estimated transmission flow ratios, based on data excluding same-sex pairs *** (mean, 95% credibility interval of posterior density)
**Source**	**Recipient**			
Men 18–24 years	Women 18–24 years	9 (12.5%)	9.1% (4.2–15.7%)	3.16 (0.92–14.44)
Men 18–24 years	Women 25–59 years	9 (12.5%)	11.7% (5.6–19.9%)	1.29 (0.47–3.69)
Men 25–59 years	Women 18–24 years	13 (18.1 %)	17.0% (9.2–26.3%)	2.63 (0.96–8.28)
Men 25–59 years	Women 25–59 years	18 (25%)	29.8% (19.2–41.7%)	2.29 (1.02–5.52)
Women 18–24 years	Men 18–24 years	3 (4.2%)	3.1% (0.7–7.5%)	--
Women 18–24 years	Men 25–59 years	5 (6.9%)	6.7% (2.3–13.4%)	--
Women 25–59 years	Men 18–24 years	7 (9.7%)	9.2% (3.8–16.8%)	--
Women 25–59 years	Men 25–59 years	8 (11.1%)	13.4% (6.1–22.9%)	--

* Phylogenetically reconstructed transmission events, unadjusted for sequence sampling differences across sampling groups. ** Estimated transmission probabilities from a source to recipient group in the sample; shown are the mean and corresponding 95% credibility interval of posterior density. *** Estimated flow ratios were respectively M18–24->F18–24 / F18–24->M18–24 and M25–59->F18–24 / F18–24->M25–59.

**Table 5:** Phylogenetically estimated sources of HIV-1 acquisition among male and female study participants by age.

	Estimated sources of infection among study participants, based on data excluding same-sex pairs * (mean, 95% credibility interval of posterior density)			
	Men 18–24 years	Men 25–59 years	Women 18–24 years	Women 25–59 years
**Recipient**				
Women 18–24 years	34.7% (17.1–55.9%	65.3% (44.1–82.9%)	--	--
Women 25–59 years	27.7% (14.1–45.7%)	72.3% (54.3–85.9%)	--	--
Men 18–24 years	--	--	24.2% (6.3–54.0%)	75.8% (46.0–93.7%)
Men 25–59 years	--	--	32.6% (12.7–59.9%)	67.4% (40.1–87.3%)

Estimated transmission probabilities from a source to recipient location; shown are the mean and corresponding 95% credibility interval of posterior density. Rows sum to 100%.

## Supplementary Materials

The following are available online at https://www.mdpi.com/1999-4915/12/3/331/s1, Figure S1: Markov Chain Monte Carlo (MCMC) simulation trajectories for estimated transmission flows within/between populations after adjusting for sampling heterogeneity, Table S1: Mean pairwise distances in phylogenetically reconstructed transmission networks, Table S2: Phylogenetically reconstructed source–recipient pairs by location and gender.

## Appendix A

**Table A1:** PANGEA 2 Steering Committee.

Name	Institution	Representatives & Roles	Email Address
Lucie Abeler-Dörner	University of Oxford	Project manager	lucie.abeler-dorner@bdi.ox.ac.uk
Helen Ayles	PopART/Zambart	PopART	helen@zambart.org.zm
David Bonsall	University of Oxford	Sequencing lab	david.bonsall@bdi.ox.ac.uk
Rory Bowden	University of Oxford	Sequencing lab	rbowden@well.ox.ac.uk
Vincent Calvez	Institut Pasteur	TasP trial	vincent.calvez@me.com
Max Essex	Harvard Botswana	Botswana studies	messex@hsph.harvard.edu
Sarah Fidler	PopART/Imperial College London	PopART	s.fidler@imperial.ac.uk
Christophe Fraser	University of Oxford	Principal Investigator PANGEA 2, Executive Committee and PopART Phylogenetics	christophe.fraser@bdi.ox.ac.uk
Kate Grabowski	Johns Hopkins University	Executive Committee and Rakai	mgrabows@jhu.edu
Tanya Golubchik	University of Oxford	Data manager	tanya.golubchik@bdi.ox.ac.uk
Richard Hayes	PopART/LSHTM	PopART	Richard.Hayes@lshtm.ac.uk
Joshua Herbeck	University of Washington	Partners PrEP; Partners in Prevention	herbeck@uw.edu
Joseph Kagaayi	Rakai Health Sciences Program	Rakai Health Sciences Program	jkagayi@rhsp.org
Pontiano Kaleebu	MRC/UVRI Uganda	MRC studies	pontiano.kaleebu@mrcuganda.org
Jairam Lingappa	University of Washington	Partners PrEP; Partners in Prevention	lingappa@uw.edu
Sikhulile Moyo	Botswana Harvard AIDS Institute Partnership	Botswana studies	sikhulilemoyo@gmail.com
Vladimir Novitsky	Harvard University	Botswana studies	vnovi@hsph.harvard.edu
Deenan Pillay	Africa Health Research Institute (AHRI)/University College London	Principal Investigator PANGEA-1/Executive Committee, AHRI studies	dpillay@ahri.org
Thomas Quinn	Johns Hopkins University	Rakai Health Sciences Program	tquinn2@jhmi.edu
Andrew Rambaut	University of Edinburgh	Executive Committee	a.rambaut@ed.ac.uk
Oliver Ratmann	Imperial College London	Analysis	oliver.ratmann@imperial.ac.uk
Janet Seeley	MRC/UVRI Uganda/LSHTM	MRC Uganda	Janet.Seeley@LSHTM.ac.uk
Deogratius Ssemwanga	MRC/UVRI Uganda	MRC studies	Deogratius.Ssemwanga@mrcuganda.org
Frank Tanser	Africa Health Research Institute	AHRI studies	ftanser@lincoln.ac.uk
Maria Wawer	Johns Hopkins University	Rakai Health Sciences Program	mwawer1@jhu.edu

## Appendix B

**Table A2:** PANGEA 1 Steering Committee.

Name	Institution	Study Team Representatives	Email Address
Myron Cohen	University of North Carolina	--------	myron_cohen@med.unc.edu
Tulio D’Oliveira	University of KwaZulu-Natal	--------	tuliodna@gmail.com
Ann Dennis	University of North Carolina	--------	ann_dennis@med.unc.edu
Max Essex	Harvard Botswana	Botswana studies	messex@hsph.harvard.edu
Sarah Fidler	PopART/Imperial College London	PopART Phylogenetics	s.fidler@imperial.ac.uk
Dan Frampton	University College London	--------	d.frampton@ucl.ac.uk
Christophe Fraser	University of Oxford	PopART Phylogenetics	christophe.fraser@well.ox.ac.uk
Tanya Golubchik	University of Oxford		tanya.golubchik@bdi.ox.ac.uk
Richard Hayes	PopART/LSHTM	PopART Phylogenetics	Richard.Hayes@lshtm.ac.uk
Josh Herbeck	University of Washington	Partners PrEP; Partners in Prevention	herbeck@uw.edu
Anne Hoppe	University College London	Project Manager PANGEA 1/EARNEST	a.hoppe@ucl.ac.uk; hoppe.anne@gmail.com
Pontiano Kaleebu	MRC/UVRI Uganda	MRC studies	pontiano.kaleebu@mrcuganda.org
Paul Kellam	Cambridge University	--------	paul.kellam@kymab.com
Cissy Kityo	EARNEST/JCRC Uganda	EARNEST	ckityo@jcrc.org.ug
Andrew Leigh-Brown	University of Edinburgh	--------	A.Leigh-Brown@ed.ac.uk
Jairam Lingappa	University of Washington	Partners PrEP; Partners in Prevention	lingappa@uw.edu
Vladimir Novitsky	Harvard University	Botswana studies	vnovi@hsph.harvard.edu
Nick Paton	EARNEST/University of Singapore	EARNEST	nick.paton@ucl.ac.uk
Deenan Pillay	Africa Health Research Institute/University College London	Principal Investigator PANGEA 1/Africa Health Research Institute studies	dpillay@ahri.org
Tom Quinn	Johns Hopkins University	Rakai Health Sciences Program	tquinn2@jhmi.edu
Oliver Ratmann	Imperial College London	--------	oliver.ratmann@imperial.ac.uk
Deogratius Ssemwanga	MRC/UVRI Uganda	MRC studies	Deogratius.Ssemwanga@mrcuganda.org
Frank Tanser	Africa Health Research Institute	--------	ftanser@gmail.com
Maria Wawer	Johns Hopkins University	Rakai Health Sciences Program	mwawer1@jhu.edu
