# Supplementary material for: Phylogenetic and Demographic Characterization of Directed HIV-1 Transmission Using Deep Sequences from High-Risk and General Population Cohorts/Groups in Uganda
Source: Viruses. 2020 Mar 18;12(3):331. doi: 10.3390/v12030331 (PMC7150763; doi:10.3390/v12030331)
Supplement: Supplementary file 1 [file viruses-12-00331-s001.pdf]

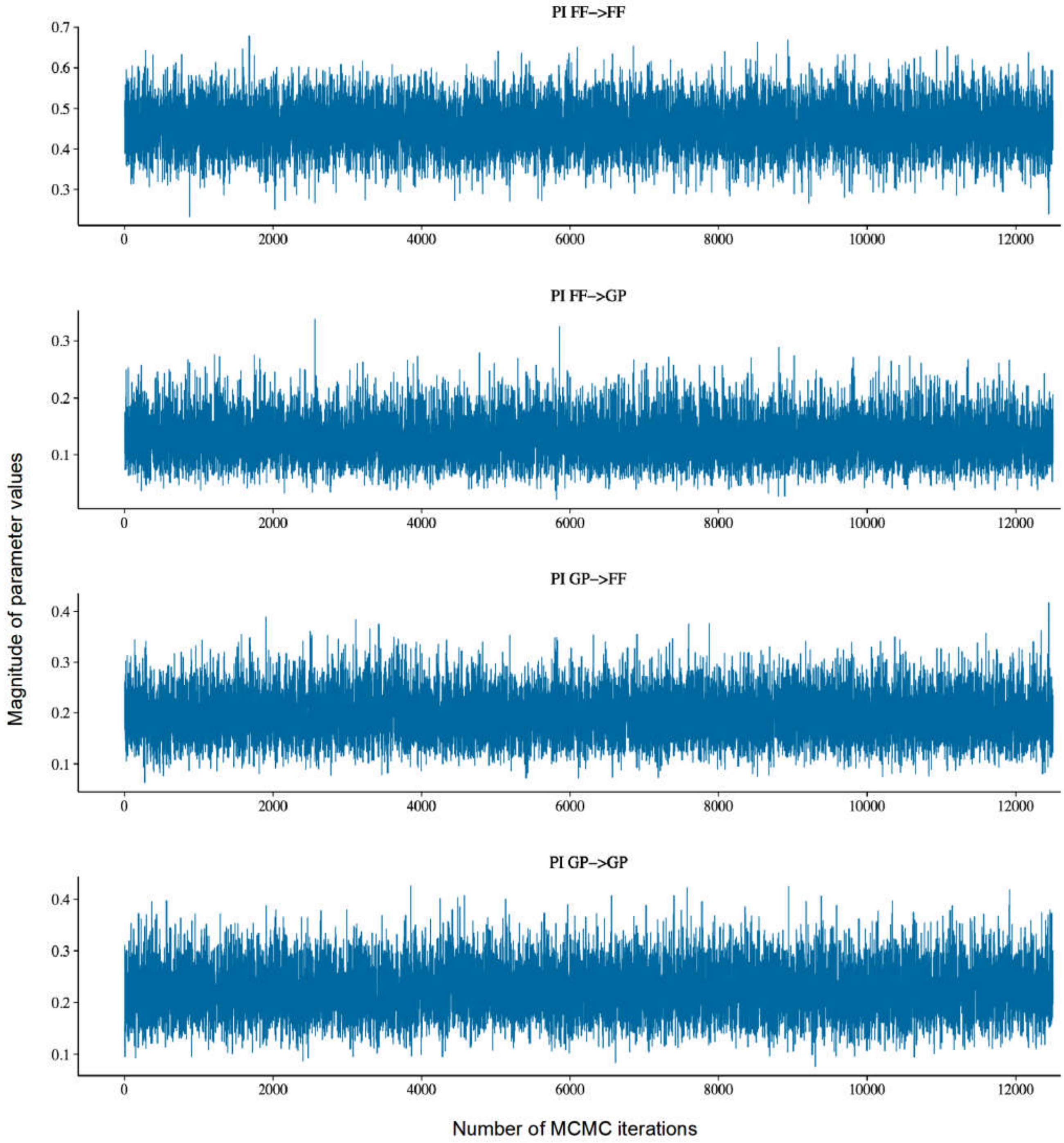

Figure S1. **Markov Chain Monte Carlo (MCMC) trace plots.** MCMC trajectories for the estimated transmission flows (PI) in the fisherfolk (FF) communities, general population (GP) and between populations after adjusting for sampling heterogeneity. The scale on the x-axis represents the number of iterations of the MCMC chain and the y-axis shows the magnitude of the parameter value that was drawn from the posterior distribution. The trajectories indicate good behavior of the algorithm in sampling from the posterior distribution of the parameter estimates reported in table 1, in terms of convergence and mixing. Trace plots of other analyses were similar.

Table S1: Mean pairwise distances in phylogenetically reconstructed transmission networks

Size of reconstructed transmission networks	Number of networks	Mean pairwise subgraph distance between individuals for whom linkage was not excluded* (in 10^{-3} mutations per site)		
		**Median**	**Lower quartile**	**Upper quartile**
**2**	101	0.5%	0.0%	1.4%
**3-5**	20	0.6%	0.3 %	1.9%
**6-10**	0	--	--	--
**>10**	2	2.1%	0.8%	2.4%

*Pairs of individuals were included in the analysis when the proportion of deep-sequence phylogenies in which the two individuals had disconnected subgraphs was below 60% (see Methods for choice of threshold). Means were calculated over deep-sequence phylogenies in which subgraphs of both individuals were adjacent.

Table S2: Phylogenetically reconstructed source-recipient pairs by location and gender

Source population	Recipient population		
	**FF, female**	**GP, female**	**WHR, female**
**FF, male**	28	2	1
**GP, male**	6	13	0
**WHR, male**	0	0	0
	**FF, female**	**GP, female**	**WHR, female**
**FF, female**	0	0	1
**GP, female**	0	8	0
**WHR, female**	0	0	10
	**FF, male**	**GP, male**	**WHR, male**
**FF, female**	5	7	0
**GP, female**	8	3	0
**WHR, female**	0	1	0
	**FF, male**	**GP, male**	**WHR, male**
**FF, male**	0	0	0
**GP, male**	0	12	0
**WHR, male**	0	0	0
